# Systems-Pharmacology Dissection of Traditional Chinese Medicine Compound Saffron Formula Reveals Multi-scale Treatment Strategy for Cardiovascular Diseases

**DOI:** 10.1038/srep19809

**Published:** 2016-01-27

**Authors:** Jianling Liu, Jiexin Mu, Chunli Zheng, Xuetong Chen, Zihu Guo, Chao Huang, Yingxue Fu, Guihua Tian, Hongcai Shang, Yonghua Wang

**Affiliations:** 1 College of Life Science, Northwest University, Xi’an, Shaanxi 710069, China; 2 College of Life Science, Northwest A & F University, Yangling, Shaanxi 712100, China; 3 Center of Bioinformatics, Northwest A & F University, Yangling, Shaanxi 712100, China; 4 Key laboratory of Chinese internal medicine of MOE and Beijing, Beijing university of Chinese medicine, Beijing, 100700, China

## Abstract

Cardiovascular diseases (CVDs) have been regarding as “the world’s first killer” of human beings in recent years owing to the striking morbidity and mortality, the involved molecular mechanisms are extremely complex and remain unclear. Traditional Chinese medicine (TCM) adheres to the aim of combating complex diseases from an integrative and holistic point of view, which has shown effectiveness in CVDs therapy. However, system-level understanding of such a mechanism of multi-scale treatment strategy for CVDs is still difficult. Here, we developed a system pharmacology approach with the purpose of revealing the underlying molecular mechanisms exemplified by a famous compound saffron formula (CSF) in treating CVDs. First, by systems ADME analysis combined with drug targeting process, 103 potential active components and their corresponding 219 direct targets were retrieved and some key interactions were further experimentally validated. Based on this, the network relationships among active components, targets and diseases were further built to uncover the pharmacological actions of the drug. Finally, a “CVDs pathway” consisted of several regulatory modules was incorporated to dissect the therapeutic effects of CSF in different pathological features-relevant biological processes. All this demonstrates CSF has multi-scale curative activity in regulating CVD-related biological processes, which provides a new potential way for modern medicine in the treatment of complex diseases.

As “the world’s first killer” of human beings, especially for middle-aged and elderly people^1^, CVDs deprives more than 10 million human lives every year, and the mortality is projected to increase to 23.6 million by 2030^2^. With different CVDs, various allopathic medicines have been produced in recent years, such as thrombolytic agents, beta-blockers, parental nitroglycerine, heparin, calcium blockers and a variety of anti-platelet agents. The significant curative effects of these medications are undoubted, but they actually cured the symptoms not the diseases and some certain unavoidable ill-effects still exist. For example, in the treatment of cardiac arrhythmias, nearly all western antiarrhythmic medications result in adverse effects at different degree, sometimes even proarrhythmic^3^.

With the rapid development of modern medical sciences, people gradually find out that most diseases are usually not caused by single target, but multiple genes^4^, the medical failures of CVDs might ascribe to the incomplete understanding of their complexity. Thus, product caters for the multi-target therapy is urgently needed in combating this ferocious killer. Traditional Chinese Medicine (TCM) has upheld the holistic therapeutic philosophy for more than 2000 years, with its increased popularity on a world wide scale in recent decades, CVDs patients begin to prefer integration of TCM and modern medicine, or fully TCM therapy^2^. However, the huge difference between modern medicine and TCM still push TCM away from the mainstream medicine.

Traditional Uygur medicine (TUM), as a significant component of TCM, has achieved notable success in CVDs prevention and treatment, with compound saffron formula (CSF) as a notable example. Similar to the other traditional Chinese formula, CSF is a complex system with the interactions among multi-components, multi-targets and multi-mechanisms. CSF is comprised of 10 herbs and 3 animal drugs, which including *Crocus sativus* (CS, Xihonghua)*, Dracocephalum moldavica* (DM, Xiangqinglan)*, Rosa rugosa* (RR, Meigui)*, Malus pumila* (MP, Pingguo), *Fructus Foeniculi* (FF, Xiaohuixiang)*, Lavandula angustifolia* (LA, Xunyicao)*, Eletteria cardamomum* (EC, Xiaodoukou)*, Nardostachys chinensis* (NC, Gansong)*, Anchusa italic* (AI, Niushecao)*, Santalum album* (SA, Tanxiang)*, Moschus* (MO, Shexiang)*, Silkworm cocoom* (SC, Canjian) *and Beaver Castoreum* (BC, Hailixiang). This formula has been applied to treat CVDs accompanied by symptoms such as arrhythmia, cardio-thoraco-algia and palpitation in Xinjiang district, and the biological effects on CVDs of drugs in this formula have also been validated in previous research. For example, Shexiang Baoxin Pill, with MO as its main element, has been proven to suppress the processes of oxidative injury and inflammation which are induced by myocardial infarction^5^. CS can ameliorate the damages of hyperlipidemia^6^. Besides, NC display well in normalizing heart rate and rhythm in people with tachyarrhythmias^2^ and LA has been demonstrated to improve the coronary flow velocity^7^.

Despite of the promising effects of TCM, how herbal formula work and what are their targets are still ambiguous. To explore the mechanism details and the relevant biological basis of CSF in the treatment of CVDs, the following numerous issues need to be solved urgently: 1) which active ingredients are involved in the regulatory processes of CSF in CVDs treatment? 2) Which targets are modulated by the active chemicals to achieve the biological activity? 3) Which pathologic processes are regulated by the active ingredients and drugs to achieve the purpose of curing CVDs? With the growing prosperity of system pharmacology nowadays, those powerful analytical tools such as network pharmacology and pharmacokinetics evaluation allow us to penetrate into the complex and holistic mechanisms of TCM in treating complex diseases^8^. Hence, in this study, we developed a system pharmacology approach to insight into the molecular mechanisms of CSF in treating CVDs. Briefly, as seen in Fig. 1, we first adhere to an *in silico* ADME system to filter out the active ingredients with favorable pharmacokinetics activity, which were then used as the baits to fish their related targets, and the reliability of drug-target interactions were experimentally validated. The obtained potential targets were then mapped onto relevant databases to find out their corresponding CVDs and pathways. Then, network construction and pathway integration analysis were performed to illustrate the molecular mechanism of CSF on CVDs holistically. We believe that the exploration of the underlying molecular mechanisms of TCM with the help of system pharmacology approach will positively promote the development of new therapy for complex diseases in the near future.

## Results

### Active components identification

Compounds satisfy the screen criteria and their corresponding drugs were presented on Supplementary Table S1.

#### Specific components of drugs in CSF

Most of the drugs possess their specific ingredients. For example, crocetin, one of the most effective constituents in CS, has been reported to possess protective effects on atherosclerosis, thrombosis, cardiac hypertrophy and hypertension due to its anti-oxidants, anti-platelet, anti-inflammatory and anti-apoptosis activity^9^,^10^,^11^. As the major component of MO, muscone plays a protective role on ischemia–reperfusion injury in cardiac myocytes owning to its antioxidant and anti-apoptosis effects^12^. Patchouli alcohol, the specific component of NC, has been confirmed to down-regulate the expression of a number of inflammatory mediators such as COX-2, IL6 and so forth, which closely associated with the process of atherosclerosis^13^. The specific components of RR are eugenol and catechin, it has been reported that eugenol has anti-inflammatory and antioxidant activities in CVDs^14^, and catechin was suggested to reduce body fat and decrease CVDs risks^15^. *In vivo* evidence has proven the inhibitory effect of cardamonin (a crucial active ingredient of EC) on angiotensin II-induced proliferation and migration in rat vascular smooth muscle cells, which is critical to the prevention of atherosclerosis^16^. Thus, this component could be considered as a curative element in treating atherosclerosis.

#### Shared components of drugs in CSF

As can be seen from Supplementary Table S1, there exist more than 20 active compounds shared by two or more drugs in CSF. For example, β-sitosterol, a common ingredients of 7 drugs such as SC, DM and AI, shows an inhibitory effect on the expression of vascular adhesion molecule 1 and intracellular adhesion molecule 1, which mediate the chronic inflammatory process involves in atherosclerosis^17^. Lycopene (CS and RR) and carotenes (CS, MP, RR and DM) have been demonstrated to prevent CVDs because of their antioxidant effects^18^,^19^,^20^. Linalyl acetate (LA and EC), as the major components of lavender essential oil, was found to relax the rabbit vascular smooth muscle^21^. Borneol, a shared component of LA and BC, possesses anticoagulant effect thus can prevent thrombosis^22^. In addition, α-pinene (NC, FF and LA) and β-pinene (NC and LA) have been suggested to be capable of reducing blood pressure in the previous research^23^.

### Target fishing and functional analysis

By means of the methods we stated in 2.3, after discarding 3 compounds with no targets, 1291 compound-target interactions were generated between 100 compounds and 219 targets. The following action mode prediction showed that 73 of these DTIs are activation patterns, the rest of them are inhibition, and many of them are verified in previous research, such as PTGS2 (Cyclooxygenase-2)^24^ and CDK6 (Cyclin-Dependent Kinase 6)^25^. In the subsequent GOBP enrichment analysis, we are delighted to find out that there are a number of targets involve in several CVDs-associated biological processes such as positive regulation of vasodilation, positive regulation of blood pressure, regulation of the force of heart contraction, positive regulation of inflammatory response and so forth (P≪0.01). The succedent functional annotation clustering analysis further summarized these biological processes into several functional modules such as enzyme synthesis and activity regulation, metabolic regulation, inflammatory regulation, cell death, regulation of vascular and muscle contractility and so forth (P≪0.01) (Supplementary Table S2).

For example, (1) Blood-borne inflammation, which belongs to the inflammatory regulation module, is ubiquitous in all stage of atherosclerotic coronary artery disease from the original lesion to the ultimate thrombotic complications^26^. AOC3 (Membrane primary amine oxidase), which is active in adipocytes and smooth muscle cells, has been validated to be an anti-inflammatory target in the process of drug development^27^. (2) Clinical data have confirmed the vital role of apoptosis (belongs to the regulation module of cell death) in the pathogenesis in a variety of CVDs, such as myocardial infarction, heart failure, and atherosclerosis, owning to the cardiac cells injury^28^, the activation of RXRA (Retinoic acid receptor RXR-alpha) has been reported to be closely related to hyperglycemia-induced cardiac myocytes apoptosis^29^. (3) The dynamic balance between vasodilation and vasoconstriction is crucial for the blood pressure (belongs to the vascular and muscular contractility module) to be maintained at the normal level. Once this balance was broken, it will interfere with the normal process of blood fluidity, platelet aggregation and smooth muscle cell proliferation^30^. *In vivo* evidence has suggested the potential effect of CALCA (Calcitonin gene-related peptide 1) in blood pressure regulation^31^.

### Ligand-target analysis

We performed ligand-binding assays for some key predicted drug-target interactions to validate the inhibitory effects of each compound on their related targets. The experimental results were listed in Table 1. Compounds kaempferol and catechin were tested in MPO inhibition assays and were proven to be potent inhibitors. At 5 μM, the highly-efficient compound catechin directly binds to MPO and decreases the activity of MPO for 86%. Compound kaempferol shows down-regulated activity of MPO for 84% at 10 μM doses. Besides, compound isorhamnetin was tested in F2 and PIM1 inhibition assays and both of them show relatively weak inhibitory activities. The activity of F2 and PIM1 reduced by 36% and 24% at 100 μM doses and 10 μM, respectively. To some extent, these results demonstrated that the drug-target interactions obtained by the theoretical prediction are reliable.

### Network construction

#### Compound-target-function (C-T-F) network

Figure 2a displays the resultant C-T-F network which is consists of 100 compounds, 219 candidate targets and their corresponding 9 functional annotations. The result displayed an average degree of 13 per compounds and 5.9 per target proteins, respectively. In the relationships between compounds and targets (Fig. 2b), for those active compounds, MOL178 exhibits the highest number of target candidate target interaction (degree = 56), followed by MOL269 (degree = 44), MOL206 (degree = 35), MOL221 (degree = 30) and so forth, these indicate the multi-target properties of ingredients, which is the essence of the action mode of herbal drugs. As for the candidate targets, TRPC4 (Short transient receptor potential channel 4) shows the highest degree (Degree = 41), with CPB2 (Carboxypeptidase B2 isoform A, Degree = 40), ENPEP (Aminopeptidase A, Degree = 38), HSD11B2 (11-beta-Hydroxysteroid Dehydrogenase 2, Degree = 31) behind it, which demonstrate the potential therapeutic effect of each drug in CXF for combating CVDs through modulating these relevant proteins.

TRPC4 involves in the progression of Ca2^+^ transport in vascular and muscular contractility regulation, recent evidence suggested that the up-regulation of TRPC4 may lead to cardiac hypertrophy and heart failure^32^, thus the suppression of TRPC4 by active ingredients such as MOL103 and MOL174 is required for cardio protection. MOL 193 and MOL221 are shown to decrease ENPEP level, and previous *in vivo* evidence unraveled that the inhibition of ENPEP reduces blood pressure^33^. HSD11B2 was shown to participate in several biological processes such as the regulation of systemic arterial blood pressure, hormone synthesis and metabolism, oxidation-reduction reaction, lipid biosynthetic process and so forth. Previous study demonstrates that the suppression HSD11B2 activity will activate mineralocorticoid receptor and increase blood pressure at last^34^, thus, the activation effect of MOL195 on HSD11B2 will definitely avoid hypertension and reduce the risk of CVDs at the same time. In addition, MOL222 serves as an inhibitor of CPB2, and elevated plasma CPB2 concentration was proven to induce venous thrombosis^35^.

#### Target-CVDs network

In this section, to illustrate the peculiar action mechanisms of the potential target case by case, relevant CVDs and their associated targets were constructed into a target-CVDs (T-cD) network.

As shown in Fig. 3, *in vivo* evidence indicated that over-activating of NOX4 (NADPH oxidase 4) aggravated the impairment of cardiac function and apoptosis under overpressure condition^36^. GSK3B (Glycogen Synthase Kinase-3, beta) was reported to prohibit mitochondrial permeability transition pore opening, which acts as the inducement of cell necrosis and myocytes apoptosis^37^. Thus, modulations of compounds like MOL178 and MOL269 are considered to be a promising therapeutic plan in myocardial protection in heart failure. As one of the main risk factors of CVDs, hypertension links with more than twenty targets in this T-cD network. EDNRA (Endothelin receptor ET-A) and NR3C2 (Mineralocorticoid receptor) were predicted to be suppressed by MOL82 and MOL195, respectively, and clinical data found overexpression of EDNRA in arteries of hyperpietic in previous research. Besides, protein NR3C2 (Mineralocorticoid receptor) was also reported being an influencing factor in blood pressure regulation owing to the promote action on salt retention in kidney^38^. Hence, the inhibition of these targets deserves more attention in subsequent therapy. All of the above statement demonstrate the multi-target therapeutic efficiency of CSF in the treatment of CVDs.

Meanwhile, the occurrence of CVDs is multi-factorial, mutual influences exist between different kinds of diseases, thus the shared target proteins of them would be the potentially valuable therapeutic targets in the treatment of CVDs from an overall standpoint. For example, PTGS2 (Cyclooxygenase-2) is one of the risk factors in the morbidity and prognosis of CVDs, its product Prostaglandin E2 involved in the inflammation processes in vascular smooth muscle cells^39^. This target is related to 22 CVDs-associated diseases such as thrombosis, chronic kidney failure, cardiomyopathies, atherosclerosis, diabetes mellitus and obesity in T-D network. Clinical research demonstrated that patients with type 1 diabetes exhibit increased expression of PTGS2 in the peripheral blood mononuclear cells (PBMCs), which leads to dysfunction of PBMCs^40^. In addition, enhanced PTGS2 protein level was also detected in PBMCs and atherosclerotic plaques in atherosclerotic relative to healthy individuals^39^. Thus, the inhibition of PTGS2 is essential for the restoration of PBMCs activity and in the process of atherosclerosis. In our study, MOL178 was predicted to decrease PTGS2 protein level, which will exert curative action on these CVDs-associated diseases.

### Pathway analysis

In this section, pathways directly related to CVDs were assembled into a “CVDs pathway” based on the present cognition of CVDs pathology. Those target proteins exhibit incredibly functional connection closeness to the proteins linked with CVDs pathway (ultimate nearness = 0.0481, nearness = 0.0027, p < 0.01). As can be seen from Fig. 4, this CVDs-associated pathway can be decomposed into several functional modules such as inflammation, myocardial contraction, DNA repair, protein synthesis, angiogenesis, aggregation, contraction, relaxation and so forth. Here, we mainly focused on four representative modules to dissect the underlying therapeutic effects of the CSF.

#### Blood pressure regulation module

Hypertension is one of the primary culprits of the high risk of many CVDs. The sustained rise in arterial pressure will ultimately lead to the impairment of a target organ such as heart and kidney and attended by systemic metabolic abnormalities. Hence, blood pressure to be controlled within the normal range through the regulation of vascular smooth muscle contraction is undoubtedly a therapeutic program in CVDs treatment. As can be seen from Fig. 4, target genes be marked in the vascular smooth muscle contraction pathway are detected to connect with blood pressure regulation. For instance, MOL170 was observed to decrease EDNRA (Endothelin-1 receptor) level, while previous clinical research indicated that down-regulation of EDNRA significantly reduced blood pressure in hypertension patients^41^. Besides, adrenergic signaling in cardio myocytes operates the function of myocardial contraction through the regulation of some certain active components on their corresponding target proteins. As displayed in Fig. 4, For example, MOL206 was predicted to decrease the activity of CAMK2D (CaM kinase II subunit delta). *In vivo* experimental data suggested that the knockout of CAMK2D shows preventive effect of cardiac hypertrophy and promoting myocardial function^42^, which is essential for the protection of cardiac contractility. In addition, MOL148 serves as the inhibitor of PRKCA in this study. PRKCA was identified as a proximal regulator of cardiac contractility and Ca2^+^ handling in cardiomyocyte, and experimental evidence demonstrated that overexpression of PRKCA hinders heart muscle contraction, whereas mouse deleted of PRKCA enhances Ca2^+^ transients and promotes cardiac contractility^43^. As stated above, the regulation of cardiac contractility-associated pathway through these target proteins is a worthwhile avenue for CVDs therapy. All these indicated that the modulation of targets in blood pressure associate pathway is a potent therapeutic mode in CVDs treatment.

#### Platelet aggregation module

Many cardiovascular diseases such as atherosclerosis, hypertension and thrombotic diseases are found to closely related to primary or secondary endothelial cell injury, which leads to vascular trauma-caused hemorrhage and subsequent disequilibrium in vasoactive substances releasing. Under this circumstance, hemostasis at the site of vascular injury through platelets aggregation is the primary remedial measure for the process of vascular repair. However, this blood coagulation becomes meaningless in the case of vascular injury with no rupture and bleeding, which impels thrombosis and leads circulatory embarrassment. As displayed in Fig. 4, target proteins modulated by active components through operating Platelet activation pathway involve in vascular repair and thrombosis. MOL203, MOL222 and MOL238 was predicted to lower PTGS1 (Cyclooxygenase-1) level, and previous study suggested that PTGS1 is related to some hemorrhagic complications because it interfered the aggregation of platelet^44^, which demonstrates that the herbal drugs modulate platelet aggregation in vascular repair process through inhibiting the activity of PTGS1. ITGA2B (Integrin alpha-IIb) was reported to aggravate the arterial thrombosis in anti-phospholipid syndrome patients^45^. Thus the inhibitory effect of MOL281 on ITGA2B will serve as an anti-thrombotic modulator in this situation. In addition, P2RY12 (P2Y purinoceptor 12), the platelet ADP receptor which was suppressed by MOL312 in our study, was always considered to be the target protein of antithrombotic drugs in clinical research^46^.

#### Angiogenesis module

Sustained angiogenesis is deemed to be an efficient therapeutic protocol in many cardiac ischemia-induced CVDs. It effectively repairs the damaged tissue and bring the blood supply to the heart back to normal. As can be seen from Fig. 4, target proteins involve in the PI3K-Akt signaling pathway are regulated by the herbal ingredients to achieve the modulation effect of angiogenesis. MOL178 and MOL170 were observed to increase GSK3B level and reduce F2R (Proteinase activated receptor 1) level, respectively. Previous *in vivo* data found out that the up-regulation of GSK3B prohibit the process of angiogenesis, whereas reduced expression of GSK3B promotes the formation of capillary^47^. Animal model evidence demonstrated that activating F2R enhances the expression of VEGF and promote angiogenesis, which certified the significant role of F2R in blood vessel recruitment^48^. All these present one of the multitudinous treatment approaches of CSF in CVDs through promoting angiogenesis.

#### Cross-talk

Biological cross-talk refers to one or more target genes of one signaling pathway affect(s) another one or more pathway(s) at the same time^49^. As we can see from Fig. 4, there exist two typical cross-talk between vascular smooth muscle contraction pathway (red) and platelet activation pathway (yellow)/PI3K-AKT signaling pathway (purple), owning to the mutual interaction between them which is implemented through the regulation of the common target protein involving in either pathway.

For example, vascular smooth muscle contraction pathway and platelet activation pathway in the schematic diagram are shown to band together to regulate intracellular signaling cascades through the management of protein PKACA (cAMP-dependent protein kinase catalytic subunit alpha). Activation of ADCY1 (adenylate cyclase 1) induces elevating the level of cAMP, which results in activation of PKACA and subsequently up-regulates VASP (Vasodilator-stimulated phosphoprotein) expression. This further activate a series of target proteins in platelet activation pathway, and ultimately leading to the aggregation of platelet. Thus, the inhibition effect of compounds such as MOL110 in LA., MOL195 and MOL339 in CS on ADCY1 will suppress the activity of PKA and the following target proteins, which hinder the aggregation of platelet, prevents thrombosis. In addition, regulatory effect of PRKCA by MOL148 from NC may also affect the cross-talk between vascular smooth muscle contraction pathway and PI3K-AKT signaling pathway, modulating the vascular and myocardial contractility through the regulation of the expression of related genes.

## Discussion

The prevalence of CVDs and the powerlessness of current western allopathic therapy in coping with complex-trait disorder makes it an urgent need for us to dig for a novel and efficient curative system from a fresh angle. The efficacy of TCM has been verified through thousands of years’ practice. Even though pharmaceutical ingredients of drugs have been extracted and purified for new drug discovery in recent years, this way always end up in failure owning to the breaking of functional rule of drugs. TCM treating complex diseases can be considered as a complexity confront another complexity, which mainly focuses on the equilibrium of the whole body through regulating the interactions among all elements within the organisms. However, the exact action mechanisms of herbal drugs on the protein and pathway level are remaining a hard nut to crack for us. Thus, in this study, we developed a systems pharmacology approach to unearth the mechanisms of CSF in CVDs treatment from a molecular to system-level.

In our work, with the help of the ADME evaluation system, 103 active compounds were identified, 100 of which could interact with 219 direct targets by drug targeting. It was found that some biological activity against CVDs of these active compounds have been reported previously^2^,^5^,^6^,^7^, highlighting the credibility of our ADME evaluation system. Then, an in house PreAM model was applied to get straight the drug-target interactions, with the GOBP clustering analysis right after it. The analytical results distinctly explained to us the action mode and biological processes that drugs utilized to achieve their curative effects. Finally, a T-cD network and a CVDs-pathway were constructed to further dissect the therapeutic polypharmacology of CSF.

Previous studies clarified that even if the “single-target” drugs exert their utmost suppression effects on their direct targets, they might not always achieve desirable results^50^. Different drugs acting on the same targets or multiple targets hit by the same drugs gain their more binding opportunity with each other and get their more chances to affecting the whole equilibrium of networks, which make the TCM therapy more effective^51^. The analytical result of the C-T network displayed an average degree of 13 per compounds and 5.9 per target proteins, respectively. Among the 101 compounds with corresponding targets, 99 were capable of acting on more than 2 targets and 44 linked with more than 13 target proteins. TLR4 (Toll-like receptor 4), for example, which is indicated to mediate vascular inflammation process^52^, was shown to be a potential therapeutic target for acute coronary syndrome, angina and atherosclerosis. It has been demonstrated that acute coronary syndrome are usually the result of the atherosclerotic plague rupture that mechanistically associated with inflammatory progression^53^. TLR4 was shown to involve in Toll-like receptor signaling pathway, PI3K-Akt signaling pathway and NF-kappa B signaling pathway in pathway analysis. Thus, in this little complex system, MOL137, MOL152, MOL178 and MOL284 which was predicted to inhibit the activity of TLR4, exert their therapeutic effects on CVDs through the regulation of inflammation-associated pathways such as Toll-like receptor signaling pathway. Hence, in this complex system with multi-drug-target-disease interactions, active ingredients will exert their different biological effects through regulating the related targets and pathways and achieve the curative results on various pathological processes of CVDs.

CVDs are not the cause of a single factor, different types of CVDs influence and aggravate each other. Pathological processes involve in the development of CVDs are complex, but medicines direct at these conditions simultaneously hasn’t appeared yet. Thus, the multi-scale curative effects of CSF through the regulation of diverse physiological functions such as blood pressure response, blood flow velocities, cardiac muscle contractility and blood-borne inflammation confirmed its excellent efficacy in combating CVDs from a panorama view. Since modern medicine unable to make a breakthrough in CVDs treatment, why not make a wise choice to shift our attention to TCM which display well in confronting complex diseases?

## Methods

**Figure 1:**
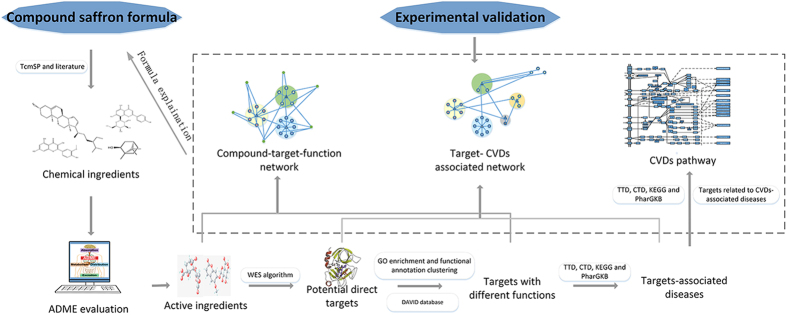
Workflow for CSF in treating CVDs.

**Figure 2:**
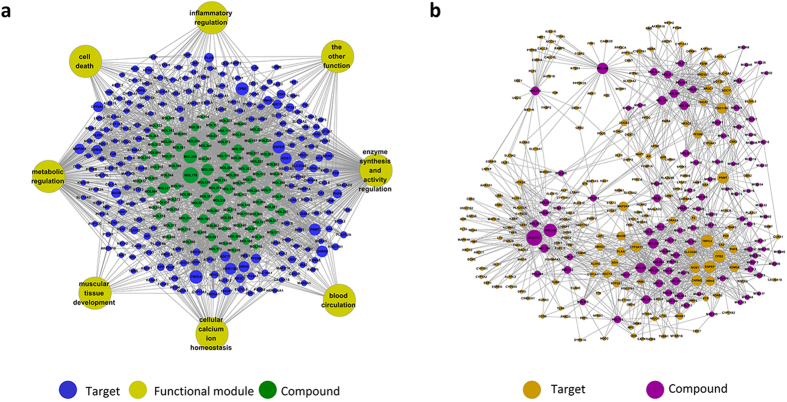
(**a**) Compound-target-function network. A compound and a target are linked if the target protein is hit by the corresponding compound. Similarly, a target and a functional module are linked if the target is involved in this biological process. Node size is proportional to its degree and the letters are node labels. (**b**) Compound-target relationship. A compound and a target are linked if the target protein is hit by the corresponding compound. Node size is proportional to its degree and the letters are node labels.

**Figure 3:**
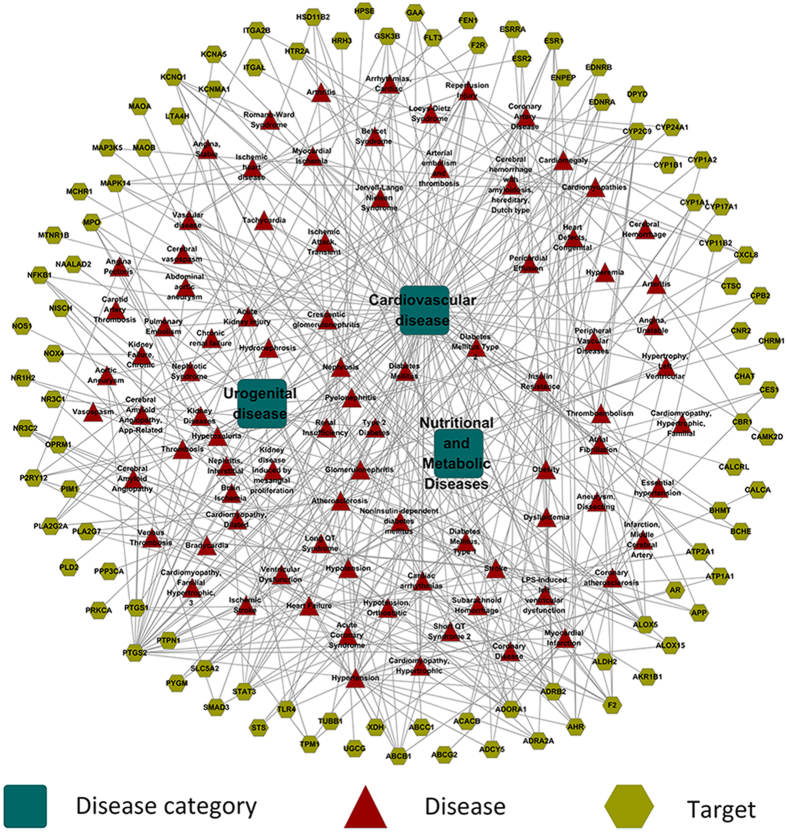
Target-CVDs associated disease network. Target proteins are linked with their corresponding diseases and those diseases are linked with the corresponding disease categories they belong to. The letters are node labels.

**Figure 4:**
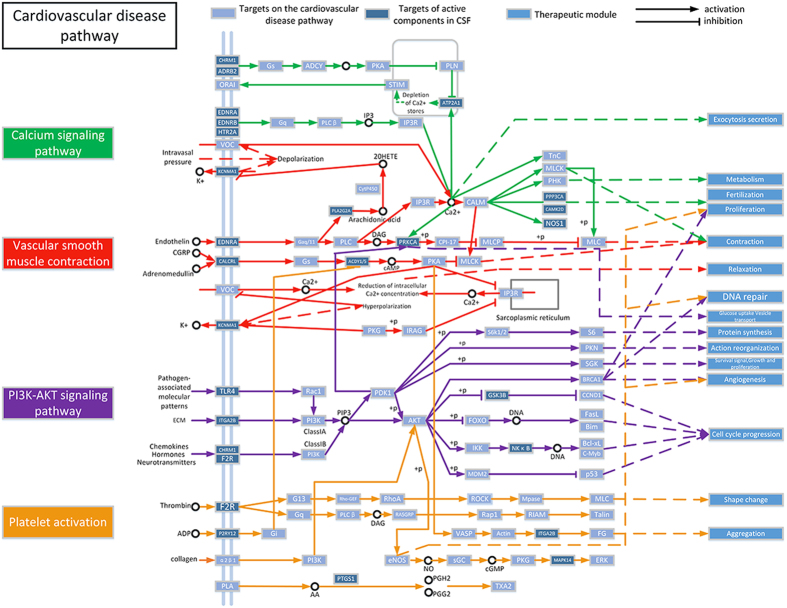
CVDs pathway and therapeutic modules.

An integrated system-based pharmacology approach has been introduced in this work to disclose the curative effects of CSF (Fig. 1), which includes: (1) molecular database construction for all 13 drugs in the formula; (2) ADME system evaluation to screen the active ingredients out of the above compound database; (3) target-fishing and functional analysis to identify the compound-target-function correlation; (4) experimental validation for drug-target interactions. (5) network construction and analysis to illustrate the molecular mechanism of CSF in treating CVDs. (6) pathway integration analysis to reveal the regulatory mode of target proteins in several functional modules from a signaling pathway level.

### Molecular database construction

A total of 375 chemical ingredients of 13 drugs in CSF were manually collected from literature and our previously developed database: Traditional Chinese Medicine Systems Pharmacology Database (TCMSP) (http://lsp.nwsuaf.edu.cn/)^54^.

### ADME evaluation

In this section, we brought up an *in silico* integrative model—ADME (absorption, distribution, metabolism and excretion of drugs) to select out the compounds with favorable pharmacokinetics properties. This ADME system including PreOB (predict oral bioavailability), PreDHL (predict drug half-life) and PreDL (predict drug-likeness).

#### PreOB

Considering that OB is one of the most desirable attributes of new drugs which represents the ratio of the orally-administered dose that reaches the circulation system with their activity unchanged, we introduced a robust in house system, OBioavail1.1^55^, to predict the OB value for drugs, which has been proven to be efficient in active ingredients screening in many previous drug screening researches.

#### PreDHL

Drug half-life means the time spent for compounds taken in by the body to fall by half, which are undoubtedly another important property for new drug discovery. PreDHL is a novel *in silico* model that enables us to predict long or short half-life of drugs through the calculation by the C-partial least square (C-PLS) algorithm at this point^56^, DHL ≥4 is indicated by “Long” while DHL <4 is expressed by “Short”.

#### PreDL

Drug-likeness of molecules is one of the contributory factors in the absorption, distribution, metabolism and elimination in human body, thus a PreDL model was developed to calculating the DL values of each active ingredients through evaluating the Tanimoto similarity^57^ between ingredients and chemicals in the Drugbank database^58^.

In this study, the threshold values for our ADME evaluation system are OB ≥30%; DL ≥0.18; HL ≥4 (long). In precondition of satisfying the OB criteria, molecules meet either DL or HL threshold value were chosen as the bioactive ingredients for further research.

### Target fishing and validation

#### Drug-targeting

Drug-targeting was implemented by a novel computational model which is designed to detect drug direct targets on the basis of a in house weighted ensemble similarity (WES) method^59^. This is a well-performed computational model in the prediction of the binding (average sensitivity 72%, SEN) and the non-binding (average specificity 82%, SPE) patterns, with the average areas under the receiver operating curves (ROC, AUC) of 85.2% and an average concordance of 77.5%. Obtained targets were subsequently sent to Uniprot (http://www.uniprot.org)^60^ to normalize their name and organisms. Only the targets of Homo sapiens were reserved for further analysis.

#### PreAM: predict mode of action for drugs

In this section, a novel PreAM model was introduced in our work to get straight the drug-target interactions (DTIs)^61^, with the overall accuracy, activated prediction accuracy and inhibited prediction accuracy of 97.3%, 87.7% and 99.8%, respectively.

#### GO enrichment and clustering analysis for targets

To find out the targets associated with the physiological features of CVDs, a Gene Ontology (GO) enrichment analysis was performed through connecting targets to DAVID (http://david.abcc.ncifcrf.gov)^62^ for classification. In this section, the GOBP (biological process) analysis result was highlighted. Then, functional annotation clustering analysis was carried out based on the GOBP enrichment data in order to further summarize these biological processes into several modules that exert different physiological functions. We only selected the terms with P value less than 0.05.

### Experimental validation

In order to validate the accuracy and efficiency of the WES method, the ligand-binding assays were performed to quantify the inhibitory effects of drugs on their predicted direct targets according to the manufacturer’s instructions. We selected 4 key and commercially available targets. Targets MPO, PIM1 and F2 were purchased from abcam, CycLex and AnaSpec, respectively. The purities of catechin and isorhamnetin and kaempferol in each sample were higher than 98% and were purchased from Yitai Technology Ltd. (Wuhan, China). All drugs were dissolved in DMS for use and freshly prepared in use to avoid loss of function under long-term storage.

### Network construction

#### Compound-target-function (C-T-F) network

In this section, a C-T-F network was constructed to expound the multi-target and multi-function therapeutic feature of the active compounds in combating CVDs. In this network, a candidate compound and a potential target protein were linked if the protein is targeted by the compound. Meanwhile, targets perform different biological processes were connected to their corresponding functional modules.

#### Target-disease (T-D) network

To explore comprehensively the interrelationship between potential targets and diseases which will help us select out those specific targets correlated with CVDs, the corresponding diseases of potential targets were extracted from TCMSP. Finally, in line with the previously obtained target-associated disease information, a T-cD network were built by linking target proteins together with their relevant diseases.

Visualization of all networks were implemented by Cytoscape 2.8.1^63^, and the quantitative property “degree” of these networks were analyzed by Network Analysis plugin and CentiScaPe 1.2 of Cytoscape^64^. In the resultant networks, compounds, targets and diseases were represented by nodes while edges indicate the interactions between them.

### Pathway constructions and analysis

To probe into the biological effects of cellular targets affect the diseases through modulating specific pathways, an incorporated “CVDs pathway” was integrated in light of present cognition of CVDs pathology. In brief, the obtained target proteins were firstly mapped to KEGG to distribute them to several pathways. Next, pathways closely related to CVDs were picked out and consolidated into a “CVDs pathway” under the pathological and clinical data. Then, a nearness analysis was performed to explore the correlativity between the “CVDs pathway” associated proteins and the obtained targets in the protein-protein interaction (PPI) network using the φ_*pp’*_ expression in HmSP^49^.

## Additional Information

**How to cite this article**: Liu, J. *et al.* Systems-Pharmacology Dissection of Traditional Chinese Medicine Compound Saffron Formula Reveals Multi-scale Treatment Strategy for Cardiovascular Diseases. *Sci. Rep.* **6**, 19809; doi: 10.1038/srep19809 (2016).

## Supplementary Material

Supplementary Information

## Figures and Tables

**Table 1:** Inhibitory rate for the selected key drug-target interactions.

NO.	Target Gene Name	Drug Name	Dosage (μM)	Inhibitory Rate (%)
1	MPO	kaempferol	10	84
2	MPO	catechin	5	86
3	F2	isorhamnetin	100	36
4	PIM1	isorhamnetin	10	24
