# Supplementary material for: Systems-Pharmacology Dissection of Traditional Chinese Medicine Compound Saffron Formula Reveals Multi-scale Treatment Strategy for Cardiovascular Diseases
Source: Sci Rep. 2016 Jan 27;6:19809. doi: 10.1038/srep19809 (PMC4728400; doi:10.1038/srep19809)
Supplement: Supplementary Information [file srep19809-s1.pdf]

Title:

Systems-Pharmacology Dissection of Traditional Chinese Medicine Compound Saffron Formula Reveals Multi-scale Treatment Strategy for Cardiovascular Diseases

Jianling Liu[1,2,3]†, Jiexin Mu[1,2,3]†, Chunli Zheng[2,3], Xuetong Chen[2,3], Zihu Guo[2,3], Chao Huang[2,3], Yingxue Fu[2,3], Guihua Tian[4], Hongcai Shang[4]*, Yonghua Wang[2,3*]

† Equal contributors

1 College of Life Science, Northwest University, Xi’an, Shaanxi 710069, China

2 College of Life Science, Northwest A & F University, Yangling, Shaanxi 712100, China

3 Center of Bioinformatics, Northwest A & F University, Yangling, Shaanxi 712100, China

4 Key laboratory of Chinese internal medicine of MOE and Beijing, Beijing university of Chinese medicine, Beijing, 100700, China

*Corresponding author: shanghongcai@126.com (H Shang) yh_wang@nwsuaf.edu.cn (Y Wang)

Table S1. Information for 103 candidate compounds in Compound Saffron formula, including their OB, DL, HL, degree and structures.

No.	Molecular ID	Compound	Herb	OB	DL	HL	Degree	Structure
1	M002	(3E)-4-[(1R,6S)-2,2,6-Trimethyl-7-oxabicyclo[4.1.0]hept-1-yl]-3-buten-2-one	CS.	30.4	0.09	L	10	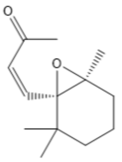
2	M004	2,6-Dimethoxy-4-methylphenol	BC.	49.2	0.04	L	14	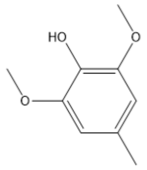
3	M008	3-carene	NC.	45.3	0.04	L	2	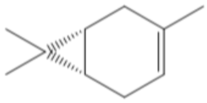
4	M021	Anisketone	FF.	39.3	0.04	L	22	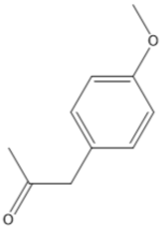
5	M029	Heptan	RR.	41.8	0.004	L	7	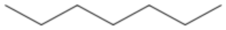
6	M030	L-Limonene	NC.<br>SA.<br>FF.	40.3	0.02	L	10	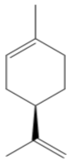
7	M031	Methyl carvacrol	NC.	55.7	0.04	L	14	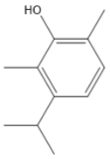
8	M046	Santenone alcohol	SA.	95	0.04	L	1	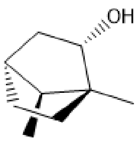
9	M048	Teresantalic acid	SA.	41.4	0.09	L	5	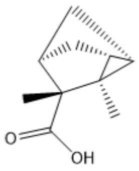
10	M051	androst-4-en-3,17-dione	MO.	39.2	0.35	S	17	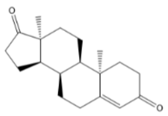

No.	Molecular ID	Compound	Herb	OB	DL	HL	Degree	Structure
11	M052	anthocyanins	MP. CS.	40.6	0.11	L	--	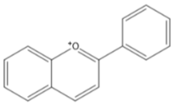
12	M053	cis-linalool oxide	BC.	65.4	0.04	L	6	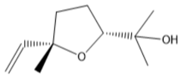
13	M068	santalone	SA.	55.6	0.09	L	3	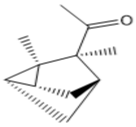
14	M077	β-maaliene	NC.	54.2	0.11	L	13	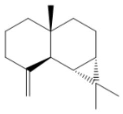
15	M078	(-)-nopinene	FF.	44.7	0.05	L	3	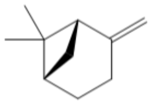
16	M082	1,8-Cineole	NC. EC. FF. LA.	39.7	0.05	L	4	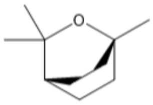
17	M090	3-methylhexane	RR.	44.9	0.01	L	6	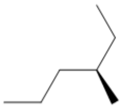
18	M091	4-Ethylguaiacol	BC.	58.3	0.03	L	16	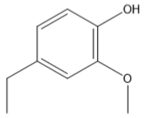
19	M093	4-Methylguaiacol	BC.	65.5	0.03	L	17	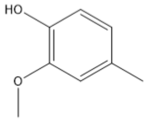
20	M094	4-Propylguaiacol	BC.	47.1	0.04	L	25	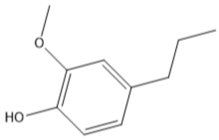
21	M100	Actinidine	NC.	71.3	0.04	L	12	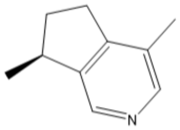
22	M102	Alpha-fenchene	LA.	41.1	0.04	L	2	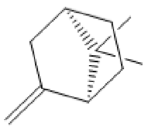
23	M103	Alpha-terpineol	NC. EC. LA.	38.7	0.03	L	11	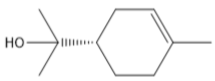
24	M104	Alpha-thujene	LA.	47.3	0.04	L	1	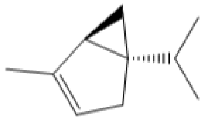

No.	Molecular ID	Compound	Herb	OB	DL	HL	Degree	Structure
25	M107	Aromadendrene, dehydro	NC.	52	0.1	L	8	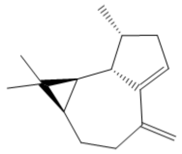
26	M108	Aromadendrene	LA.	55	0.1	L	13	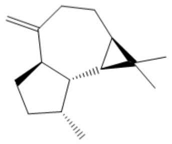
27	M109	Beta-caryophyllene	LA.	30.4	0.09	L	7	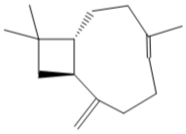
28	M110	Bornyl acetate	LA.	58.2	0.08	L	7	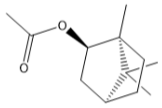
29	M116	Delta-3-carene	LA.	45.2	0.04	L	2	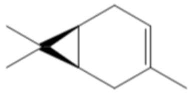
30	M117	Demethoxycapillarisin	RR.	34.9	0.25	L	15	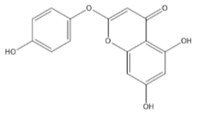
31	M118	Dipentene	FF.	39.4	0.02	L	10	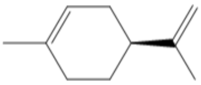
32	M119	Estragole	FF.	57.7	0.03	L	14	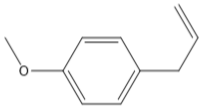
33	M121	Fenchyl acetate	FF.	100	0.07	L	10	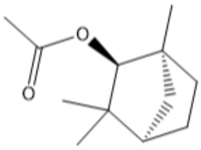
34	M122	Foron	CS.	32.8	0.02	L	11	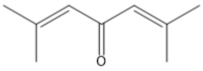
35	M123	Gamma-terpinene	LA.	33	0.02	L	8	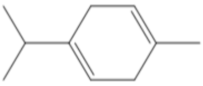
36	M133	Jatamansin	NC.	72.9	0.37	L	7	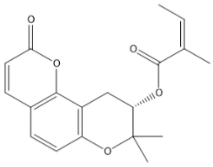
37	M136	Leaf acetate	RR.	40.3	0.02	L	10	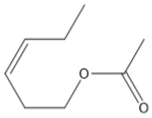
38	M137	Majudin	SA.<br>FF.	34.8	0.13	L	15	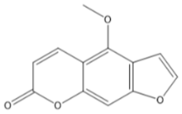
39	M143	Methyl eugenol	RR.	57.1	0.04	L	21	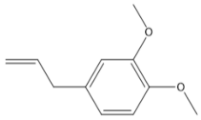

No.	Molecular ID	Compound	Herb	OB	DL	HL	Degree	Structure
40	M144	Moslene	NC.	33	0.02	L	8	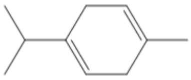
41	M148	Nardostachin	NC.	48.9	0.33	S	8	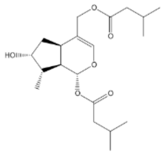
42	M151	O-Methylthymol	NC.	43.1	0.04	L	12	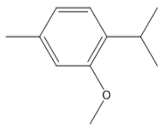
43	M152	Oroselol	NC.	53.8	0.18	L	14	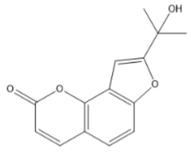
44	M155	Pymadin	SA.	51.1	0.01	L	9	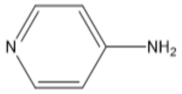
45	M157	Santene	SA.	43.1	0.03	L	3	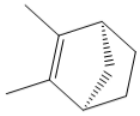
46	M158	Santenone	SA.	88.4	0.04	L	1	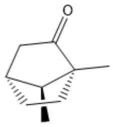
47	M160	Sulcatone	RR.	36.1	0.01	L	15	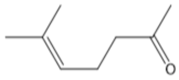
48	M161	Syringaldehyde	SA.	73.8	0.05	L	17	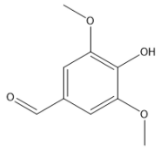
49	M167	Yomogi alcohol	NC.	38.1	0.02	L	5	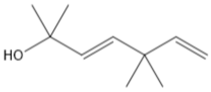
50	M170	acaciin	NC.	39.8	0.71	L	24	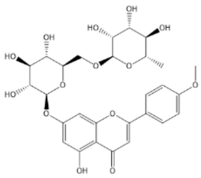
51	M173	alpha-amyrin	FF.	39.5	0.76	L	15	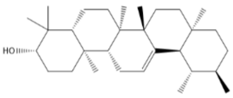
52	M174	anethole	FF.	60	0.03	L	20	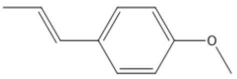
53	M175	angelicin	NC.	34.4	0.1	L	18	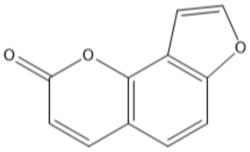

No.	Molecular ID	Compound	Herb	OB	DL	HL	Degree	Structure
54	M178	apigenin	RR.<br>DM.	61.9	0.21	L	56	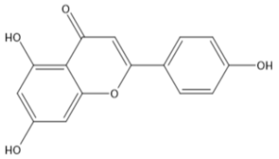
55	M188	beta-Phorone	CS.	45.6	0.03	L	6	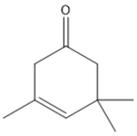
56	M192	borneol	BC.<br>LA.	83.5	0.05	L	3	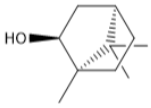
57	M193	butylated hydroxytoluene	SA.	40.6	0.07	L	8	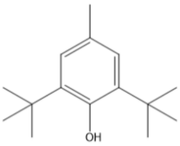
58	M195	campesterol	CS.	37.6	0.71	S	23	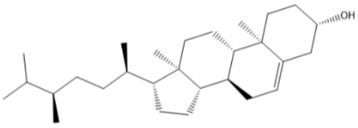
59	M196	camphene	LA.<br>FF.	36.2	0.04	L		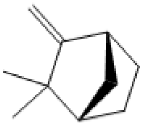
60	M198	carotenes	RR.<br>MP.<br>CS.<br>DM.	36.4	0.58	L	7	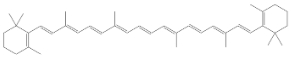
61	M200	caryophyllene oxide	LA.	34.3	0.13	L	17	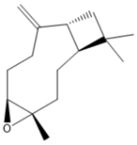
62	M203	catechin	RR.	45.8	0.24	S	18	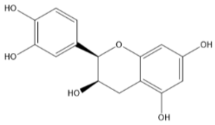
63	M206	chlorophyll A	MP.	40.7	0.1	L	35	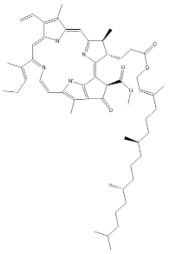
64	M208	cholest-4-ene-3-one	MO.	37.2	0.68	S	18	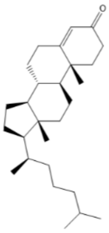

No.	Molecular ID	Compound	Herb	OB	DL	HL	Degree	Structure
65	M210	cholesterol	BC.<br>MP.<br>MO.	37.9	0.68	L	20	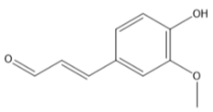
66	M221	coniferyl aldehyde	SA.	55.8	0.05	L	30	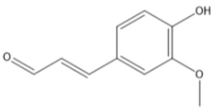
67	M222	crocetin	CS.	33.4	0.26	S	21	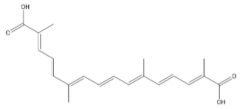
68	M224	cryptotanshinone	NC.	52.8	0.4	L	15	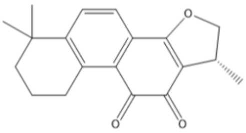
69	M226	d-Borneol	EC.	83.5	0.05	L	3	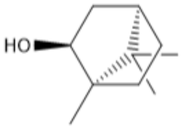
70	M228	daucene	RR.	46.4	0.08	L	7	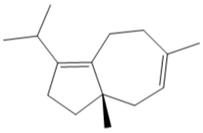
71	M238	eugenol	RR.	47.4	0.04	L	31	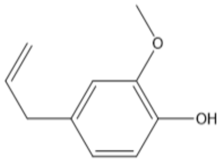
72	M240	fenchone	FF.	72.6	0.05	L	3	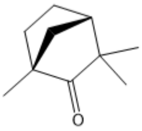
73	M258	isodaucene	RR.	47.3	0.08	L	7	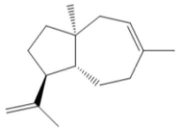
74	M259	isoeugenol	RR.	67.5	0.04	L	22	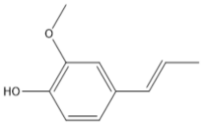
75	M264	isorhamnetin	SA.<br>CS.<br>DM.	49.6	0.77	S	26	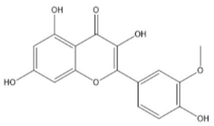
76	M266	isovitexin	SA.	69.9	0.72	S	12	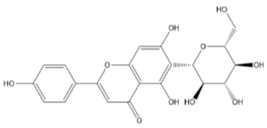

No.	Molecular ID	Compound	Herb	OB	DL	HL	Degree	Structure
77	M269	kaempferol	CS.<br>DM.	59.1	0.24	L	44	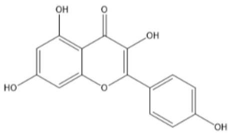
78	M273	limonene	LA.<br>EC.<br>DM.	39.4	0.02	L	10	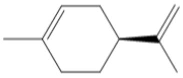
79	M275	linalyl acetate	LA.<br>EC.	42.3	0.04	L	7	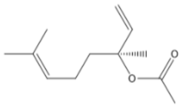
80	M281	lycopene	RR.<br>CS.	32.4	0.51	L	15	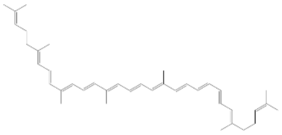
81	M284	cardamonin	EC.	34.5	0.16	L	15	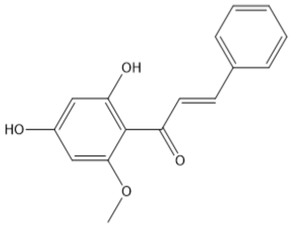
82	M287	muscone	MO.	35.7	0.37	L	9	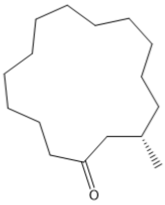
83	M288	muscopyridine	MO.	54.4	0.12	L	15	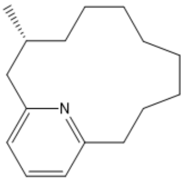
84	M301	orientin	SA.	33.1	0.18	S	17	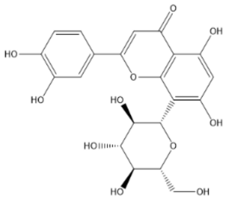
85	M307	patchouli alcohol	NC.	100	0.14	L	12	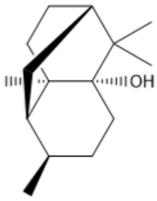
86	M312	physcion	AI.	84.8	0.27	L	20	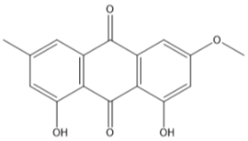

No.	Molecular ID	Compound	Herb	OB	DL	HL	Degree	Structure
87	M315	piperitone	LA.	53.9	0.03	L	9	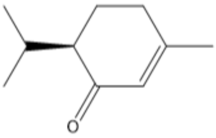
88	M317	pulegone	LA.	51.3	0.03	L	8	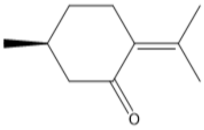
89	M323	roseoxide	RR.	37.2	0.03	L	11	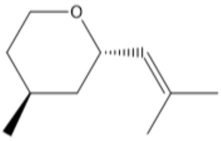
90	M332	sabinene	EC.	45.2	0.04	L	2	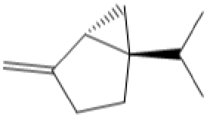
91	M337	seychellane	NC.	51.8	0.12	L	10	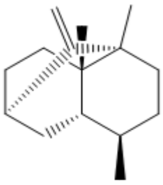
92	M338	sinapyl aldehyde	SA.	32.3	0.07	L	23	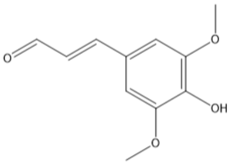
93	M339	stigmasterol	CS.<br>DM.<br>FF.	43.8	0.76	S	24	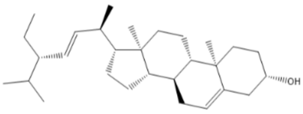
94	M344	tartaric acid	MP.<br>AI.	56	0.02	L	19	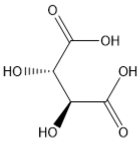
95	M346	teresantalol	SA.	31.2	0.08	L	2	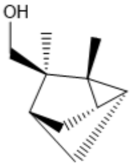
96	M348	thymol	DM.	43.3	0.03	L	13	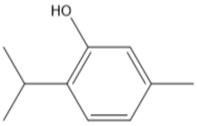
97	M354	uvqol	DM.	37.5	0.76	S	19	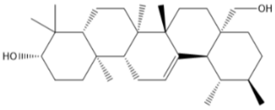
98	M357	vitexin	SA.	30.2	0.03	L	18	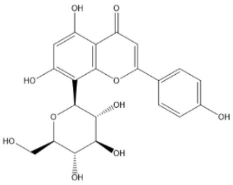

No.	Molecular ID	Compound	Herb	OB	DL	HL	Degree	Structure
99	M363	α-carotenes	CS.	37.6	0.59	L	8	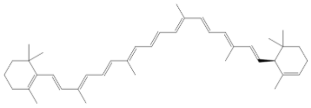
100	M365	α-pinene	NC.<br>FF.<br>LA.	46.2	0.05	L	2	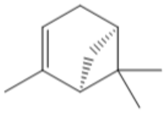
101	M372	β-myrcene	LA.<br>EC.<br>FF.	30.19	0.01	L	12	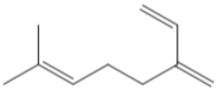
102	M373	β-pinene	NC.<br>LA.	44.7	0.05	L	3	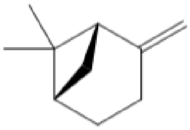
103	M374	β-sitosterol	SC.<br>NC.<br>LA.<br>RR.<br>DM.<br>FF.<br>AI.	36.9	0.75	S	25	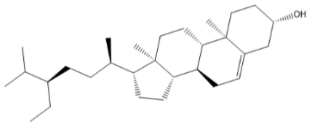

Table S2. Candidate target proteins associated biological processes.

Functional module	Biological process	Genes	Count	PValue	FDR
vascular and muscular contractility regulation	calcium ion homeostasis	PRKCA, MCHR1, DRD4, ELANE, LPAR3, FKBP1A, KCNA5, CALCA, EDNRA, EDNRB, APP, CCR6, CYP27B1, PYGM, HRH3, ATP2A1, AVPR1B, F2R	18	4.39E-09	7.65E-06
	cellular calcium ion homeostasis	PRKCA, MCHR1, DRD4, ELANE, LPAR3, FKBP1A, KCNA5, CALCA, EDNRA, EDNRB, APP, CCR6, PYGM, HRH3, AVPR1B, ATP2A1, F2R	17	2.06E-08	3.60E-05
	cytosolic calcium ion homeostasis	CALCA, EDNRA, MCHR1, EDNRB, CCR6, HRH3, AVPR1B, LPAR3, FKBP1A, KCNA5, F2R	11	1.31E-05	0.022885
	elevation of cytosolic calcium ion concentration	CALCA, EDNRA, MCHR1, EDNRB, CCR6, HRH3, AVPR1B, LPAR3, FKBP1A, F2R	10	4.72E-05	0.082367
	regulation of calcium ion transport	CALCA, EDNRA, MCHR1, ADRB2, TRPC4, NOS1, PTGS2, DRD4, MGEA5, LPAR3, FKBP1A, F2R	12	6.93E-09	1.21E-05
	negative regulation of calcium ion transport	CALCA, ADRB2, NOS1, PTGS2, DRD4	5	2.39E-05	0.041755
	positive regulation of muscle contraction	PTGS2, PTGS1, ATP2A1, SPHK1, ATP1A1, F2R	6	1.33E-05	0.023235
	regulation of smooth muscle contraction	CALCA, KCNMA1, ADRB2, PTGS2, PTGS1, SPHK1, F2R	7	2.19E-05	0.038202
	negative regulation of muscle contraction	CALCA, KCNMA1, ADRB2, ATP2A1	4	7.02E-04	1.216705
	muscle contraction	KCNMA1, EDNRA, EDNRB, GLRA1, CAMK2D, GAA, FKBP1A, CHRND, TPM1, KCNQ1, CACNA1S	11	1.22E-04	0.211774
	regulation of blood pressure	PTGS2, CYP11B2, PTGS1, ATP1A1, ADORA1, TPM1, CALCA, EDNRB, ADRB2, NISCH, HRH3, AVPR1B, HSD11B2	13	3.87E-08	6.74E-05
	regulation of systemic arterial blood pressure	CALCA, ADRB2, CYP11B2, AVPR1B, HSD11B2, TPM1	6	1.79E-04	0.31101
	positive regulation of blood pressure	CYP11B2, AVPR1B, HSD11B2, ADORA1, TPM1	5	3.75E-04	0.652636
	regulation of systemic arterial blood pressure mediated by a chemical signal	ADRB2, CYP11B2, AVPR1B, HSD11B2, TPM1	5	7.09E-04	1.229649
	regulation of vasodilation	CALCA, KCNMA1, ADRB2, NOS1, ADORA1	5	2.13E-04	0.370658
	positive regulation of vasoconstriction	PTGS2, PTGS1, ALOX5, F2R, HTR2A	5	3.42E-05	0.059587
	regulation of lipase activity	NR1H2, CALCA, GRM5, EDNRA, EDNRB, CHRM1, AVPR1B, LPAR3, FKBP1A, F2R, HTR2A	11	8.13E-07	0.001418
	phosphoinositide-mediated signaling	CALCA, GRM5, EDNRA, EDNRB, CHRM1, LPAR3, FEN1, F2R, HTR2A	9	5.91E-05	0.103093
	positive regulation of protein kinase activity	ADCY1, SPHK2, ADCY5, DRD4, ELANE, SPHK1, PIM1, FPR1, LPAR3, ADORA1, CDK5, CALCA, EDNRA, KISS1R, ADRB2, MAP3K5, CHRM1, ADRA2A, F2R, HTR2A	20	1.42E-09	2.48E-06

Functional module	Biological process	Genes	Count	PValue	FDR
	regulation of protein kinase activity	PRKCA, ADCY1, SPHK2, ADCY5, DRD4, ELANE, SPHK1, PIM1, FPR1, LPAR3, ADORA1, CDK5, CALCA, EDNRA, KISS1R, ADRB2, APP, MAP3K5, CHRM1, ADRA2A, F2R, HTR2A	22	7.46E-08	1.30E-04
	regulation of adenylate cyclase activity	OPRM1, MCHR1, ADCY1, ADCY5, DRD4, ADORA1, FSHR, CALCA, EDNRA, EDNRB, ADRB2, HRH3, GRM6, LTB4R2, ADRA2A, CALCRL	16	1.56E-11	2.72E-08
	regulation of kinase activity	PRKCA, ADCY1, SPHK2, ADCY5, DRD4, ELANE, SPHK1, PIM1, FPR1, LPAR3, ADORA1, CDK5, CALCA, EDNRA, KISS1R, ADRB2, APP, MAP3K5, CHRM1, ADRA2A, F2R, HTR2A	22	1.33E-07	2.32E-04
	positive regulation of catalytic activity	OPRM1, ADCY1, FCER2, ADCY5, DRD4, FPR1, LPAR3, ADORA1, TPM1, CALCA, SLC11A2, EDNRA, NR1H2, EDNRB, KISS1R, MAP3K5, CYP27B1, ADRA2A, CALCRL, SPHK2, SPHK1, ELANE, PIM1, SMAD3, CDK5, FSHR, PSMB8, GRM5, ADRB2, VCP, CHRM1, AVPR1B, HTR2A, F2R	34	3.01E-12	5.25E-09
	regulation of phosphorylation	ADCY1, ADCY5, DRD4, FPR1, LPAR3, FKBP1A, TLR4, ADORA1, EDNRA, CALCA, EDNRB, APP, KISS1R, MAP3K5, ADRA2A, PRKCA, SPHK2, ELANE, SPHK1, PIM1, CDK5, ADRB2, CHRM1, F2R, HTR2A	25	1.93E-07	3.37E-04
	positive regulation of kinase activity	ADCY1, SPHK2, ADCY5, DRD4, ELANE, SPHK1, PIM1, FPR1, LPAR3, ADORA1, CDK5, CALCA, EDNRA, KISS1R, ADRB2, MAP3K5, CHRM1, ADRA2A, F2R, HTR2A	20	2.57E-09	4.49E-06
	activation of protein kinase activity	CALCA, EDNRA, ADRB2, ADCY1, KISS1R, SPHK2, ADCY5, CHRM1, SPHK1, ADORA1, F2R	11	9.66E-06	0.016852
	negative regulation of cyclase activity	OPRM1, MCHR1, EDNRB, ADCY1, HRH3, ADCY5, LTB4R2, GRM6, DRD4, ADRA2A, ADORA1	11	9.09E-09	1.59E-05
	regulation of cAMP biosynthetic process	OPRM1, MCHR1, ADCY1, ADCY5, DRD4, ADORA1, FSHR, CALCA, EDNRA, EDNRB, ADRB2, HRH3, GRM6, LTB4R2, ADRA2A, CALCRL	16	3.32E-11	5.79E-08
	cAMP-mediated signaling	OPRM1, MCHR1, ADCY1, ADCY5, DRD4, FPR1, FSHR, ADORA1, P2RY12, ADRB2, GRM6, CALCRL, HTR5A	13	2.15E-08	3.75E-05
	phosphorylation	ALPPL2, ALPI, DRD4, FPR1, LPAR3, MKNK1, ADORA1, KISS1R, APP, MAP3K5, CSNK2A1, ADRA2A, CAMK2D, TNKS, PRKCA, CDC7, ERG, FLT3, ROCK2, MAP2K4, PIM1, CDK6, MAPK10, CDK5, WEE1, PRKD1, MAPK14, GSK3B, DYRK1A, MST1R, F2R	31	4.61E-06	0.008042
enzyme synthesis and activity regulation	steroid dehydrogenase activity	HSD17B2, HSD17B1, HSD11B2, SRD5A1, HSD17B3, SRD5A2	6	1.33E-04	0.198043

Functional module	Biological process	Genes	Count	PValue	FDR
	positive regulation of adenylate cyclase activity by G-protein signaling pathway	OPRM1, ADRB2, ADCY1, ADCY5, CALCRL, FSHR	6	3.40E-04	0.591715
	positive regulation of phospholipase activity	CALCA, GRM5, EDNRA, EDNRB, CHRM1, AVPR1B, LPAR3, F2R, HTR2A	9	9.86E-06	0.017196
	peptide receptor activity, G-protein coupled	OPRM1, EDNRA, MCHR1, EDNRB, KISS1R, CCR6, AVPR1B, FPR1, NTSR1, F2R	10	1.03E-04	0.152623
	protein kinase cascade	PRKCA, GRB2, DRD4, MAP2K4, FPR1, LPAR3, MKNK1, TLR4, MAPK10, ADORA1, CDK5, STAT3, P2RY12, KISS1R, MAP3K5, MAPK14, ADRA2A, F2R	18	5.48E-05	0.095582
	intracellular signaling cascade	OPRM1, MCHR1, CYP24A1, ADCY1, ADCY5, LPAR3, TLR4, ADORA1, EDNRA, EDNRB, KISS1R, MAP3K5, NISCH, RARA, CALCRL, HTR5A, PRKCA, PLD2, AR, ROCK2, RXRA, ESR1, ESR2, FSHR, CDK5, PRKD1, GRM5, ADRB2, CHRM1, GRM6, GRB2, DRD4, FPR1, MKNK1, NR3C1, NR1H2, CALCA, HRH3, CNR2, ADRA2A, FEN1, MAP2K4, SPHK1, MAPK10, STAT3, P2RY12, MAPK14, GSK3B, MTNR1B, PLA2G4B, F2R, HTR2A	52	5.22E-11	9.10E-08
	elevation of cytosolic calcium ion concentration	CALCA, EDNRA, MCHR1, EDNRB, CCR6, HRH3, AVPR1B, LPAR3, FKBP1A, F2R	10	4.72E-05	0.082367
	activation of phospholipase C activity	CALCA, GRM5, EDNRA, EDNRB, CHRM1, AVPR1B, LPAR3, F2R, HTR2A	9	6.27E-06	0.010938
	negative regulation of adenylate cyclase activity	OPRM1, MCHR1, EDNRB, ADCY1, HRH3, ADCY5, LTB4R2, GRM6, DRD4, ADRA2A, ADORA1	11	9.09E-09	1.59E-05
	inhibition of adenylate cyclase activity by G-protein signaling	OPRM1, MCHR1, ADCY1, ADCY5, GRM6, DRD4, ADORA1	7	2.97E-05	0.051777
	activation of adenylate cyclase activity	OPRM1, CALCA, EDNRA, ADRB2, ADCY1, ADCY5, CALCRL, FSHR	8	1.93E-05	0.033697
	G-protein signaling, coupled to cAMP nucleotide second messenger	OPRM1, P2RY12, MCHR1, ADRB2, ADCY1, ADCY5, GRM6, DRD4, FPR1, CALCRL, ADORA1, FSHR	12	6.55E-08	1.14E-04
	cell surface receptor linked signal transduction	OPRM1, MCHR1, ADCY1, GRIK1, GLRA1, ADCY5, LPAR3, ADORA1, EDNRA, EDNRB, KISS1R, APP, CSNK2A1, CALCRL, RAMP1, HTR5A, NTSR1, CDK5, FSHR, GRM5, ADRB2, CCR6, CHRM1, GRM6, MST1R, ITGA2B, ITGAL, ENPP2, GRB2, DRD4, FPR1, FKBP1A, CALCA, HRH3, HRH4, CNR2, ADRA2A, SPHK2, FLT3, SPHK1, SMAD3, STAT3, P2RY12, MAPK14, GSK3B, LTB4R2, AVPR1B, MTNR1B, PTPN1, HPGD, F2R, HTR2A	52	1.62E-05	0.028281

Functional module	Biological process	Genes	Count	PValue	FDR
	protein amino acid phosphorylation	DRD4, FPR1, LPAR3, MKNK1, ADORA1, KISS1R, APP, MAP3K5, CSNK2A1, CAMK2D, ADRA2A, TNKS, PRKCA, CDC7, ERG, FLT3, ROCK2, MAP2K4, PIM1, CDK6, MAPK10, CDK5, WEE1, PRKD1, MAPK14, GSK3B, DYRK1A, MST1R, F2R	29	1.15E-06	0.001998
	protein serine/threonine kinase activity	PRKCA, CDC7, ROCK2, MAP2K4, PIM1, MKNK1, CDK6, FKBP1A, MAPK10, CDK5, WEE1, PRKD1, MAP3K5, CSNK2A1, MAPK14, GSK3B, DYRK1A, CAMK2D	18	6.90E-04	1.02301
	peptide receptor activity	OPRM1, EDNRA, MCHR1, EDNRB, KISS1R, CCR6, AVPR1B, FPR1, NTSR1, F2R	10	1.03E-04	0.152623
blood circulation	blood circulation	KCNMA1, PTGS2, CYP11B2, PTGS1, ATP1A1, ADORA1, TPM1, CALCA, EDNRA, EDNRB, ADRB2, NISCH, HRH3, AVPR1B, GAA, CAMK2D, HSD11B2, KCNQ1	18	3.73E-09	6.51E-06
	oxidation reduction	XDH, ACOX1, CYP24A1, CYP1B1, PTGS2, HSD17B2, HSD17B1, CYP11B2, DUOX2, PTGS1, CYP2A13, CBR1, CYP27B1, DHCR7, HSD17B3, SRD5A1, SRD5A2, CYP19A1, NQO2, NOX4, NOS1, CYP1A1, CYP2C9, MAOA, MAOB, CYP1A2, HAO1, ALOX15, CYP17A1, KDM2A, RRM2, AKR1B10, AKR1B1, ALDH2, MPO, HSD11B2, CYP2A6, DPYD, ALOX5, HPGD, ALOX12, AOC3	42	3.05E-15	5.23E-12
	positive regulation of macromolecule metabolic process	PRKCA, KLF5, ESRRA, AR, FCER2, RXRA, ELANE, ESR1, SMAD3, TLR4, NFKB1, FKBP1A, ESR2, ADORA1, STAT3, PSMB8, AHR, TNKS2, NR1H2, EDNRA, EDNRB, ADRB2, APP, MAPK14, MGEA5, TNKS, F2R	27	6.39E-04	1.109348
	Cytochrome P450, conserved site	CYP24A1, CYP2A13, CYP17A1, CYP27B1, CYP1B1, CYP1A1, CYP2C9, CYP11B2, CYP2A6, CYP1A2, ACACB, CYP19A1	12	3.31E-10	4.83E-07
	drug metabolic process	CBR1, CYP1A1, CYP2C9, BCHE,	5	8.39E-05	0.146241
	regulation of cAMP metabolic process	OPRM1, MCHR1, ADCY1, ADCY5, DRD4, ADORA1, FSHR, CALCA, EDNRA, EDNRB, ADRB2, HRH3, GRM6, LTB4R2, ADRA2A, CALCRL	16	4.44E-11	7.74E-08
	regulation of cyclic nucleotide metabolic process	OPRM1, MCHR1, ADCY1, ADCY5, DRD4, ADORA1, FSHR, CALCA, EDNRA, EDNRB, ADRB2, HRH3, GRM6, LTB4R2, ADRA2A, CALCRL	16	1.72E-10	3.00E-07
	regulation of nucleotide metabolic process	OPRM1, MCHR1, ADCY1, ADCY5, DRD4, ADORA1, FSHR, CALCA, EDNRA, EDNRB, ADRB2, HRH3, GRM6, LTB4R2, ADRA2A, CALCRL	16	2.51E-10	4.38E-07

Functional module	Biological process	Genes	Count	PValue	FDR
metabolic regulation	phosphorus metabolic process	ALPPL2, ALPI, ENPP2, DRD4, FPR1, LPAR3, MKNK1, ADORA1, APP, KISS1R, MAP3K5, CSNK2A1, ADRA2A, CAMK2D, TNKS, PPP3CA, CDC7, PRKCA, PTPRB, ERG, FLT3, ROCK2, MAP2K4, PIM1, CDK6, MAPK10, PPP1CC, CDK5, WEE1, PRKD1, MAPK14, GSK3B, DYRK1A, PTPN1, MST1R, F2R, PPP2R2A	37	6.10E-07	0.001064
	regulation of phosphate metabolic process	ADCY1, ADCY5, DRD4, FPR1, LPAR3, FKBP1A, TLR4, ADORA1, CALCA, EDNRA, EDNRB, APP, KISS1R, MAP3K5, ADRA2A, PRKCA, SPHK2, ELANE, SPHK1, PIM1, SMAD3, CDK5, ADRB2, CHRM1, F2R, HTR2A	26	1.04E-07	1.81E-04
	icosanoid metabolic process	ACOX1, ALOX15, PTGS2, PTGS1, LTA4H, ALOX5, HPGD, PLA2G4B, ALOX12	9	4.95E-07	8.63E-04
	unsaturated fatty acid metabolic process	ACOX1, ALOX15, PTGS2, PTGS1, LTA4H, ALOX5, HPGD, PLA2G4B, ALOX12	9	9.50E-07	0.001658
	fatty acid metabolic process	HAO1, ACOX1, ALOX15, PTGS2, MAPK14, PTGS1, LTA4H, ALOX5, ELOVL6, ACACB, HPGD, PLA2G4B, ALOX12	13	5.37E-05	0.093567
	unsaturated fatty acid biosynthetic process	ALOX15, PTGS2, PTGS1, LTA4H, ALOX5, ALOX12	6	1.55E-04	0.269962
	fatty acid biosynthetic process	ALOX15, PTGS2, PTGS1, LTA4H, ELOVL6, ALOX5, ACACB, ALOX12	8	2.03E-04	0.352633
	lipid catabolic process	HAO1, ACOX1, PLD2, GBA2, CYP24A1, STS, CYP27B1, ENPP2, SPHK1, PLA2G2A, PLA2G7, SRD5A2, PLA2G4B	13	1.41E-05	0.024597
	lipid biosynthetic process	CYP1A1, SPHK2, HSD17B2, PTGS2, HSD17B1, CYP11B2, UGCG, PTGS1, ACACB, NR0B1, CYP17A1, ALOX15, DGAT1, CYP27B1, DHCR7, HSD11B2, HSD17B3, LTA4H, SRD5A1, ALOX5, ELOVL6, SRD5A2, CYP19A1, ALOX12	24	8.56E-10	1.49E-06
	steroid metabolic process	CYP24A1, STS, CYP1B1, CYP1A1, HSD17B2, HSD17B1, CYP11B2, RXRA, NR3C1, NR0B1, GBA2, CYP17A1, CYP27B1, AKR1B10, DHCR7, HSD11B2, HSD17B3, SRD5A1, SRD5A2, CYP19A1	20	2.64E-10	4.61E-07
	steroid biosynthetic process	CYP17A1, CYP27B1, HSD17B2, HSD17B1, DHCR7, CYP11B2, HSD11B2, SRD5A1, HSD17B3, SRD5A2, NR0B1, CYP19A1	12	6.55E-08	1.14E-04
	cellular hormone metabolic process	CYP17A1, CYP1B1, CYP1A1, HSD17B1, CYP11B2, HSD11B2, SRD5A1, SRD5A2, NR3C1	9	2.98E-06	0.005198
	hormone biosynthetic process	CYP17A1, HSD17B1, CYP11B2, DUOX2, HSD11B2, SRD5A1, SRD5A2	7	6.39E-06	0.011141
hormone synthesis and metabolism	hormone metabolic process	CYP17A1, CYP1B1, CYP1A1, HSD17B1, CYP11B2, DUOX2, HSD11B2, SRD5A1, SRD5A2, NR3C1	10	3.52E-05	0.061406

Functional module	Biological process	Genes	Count	PValue	FDR
inflammatory regulation	regulation of hormone levels	CYP17A1, CYP1B1, DGAT1, CYP1A1, HSD17B1, CYP11B2, DUOX2, HSD11B2, SRD5A1, SRD5A2, NR3C1	11	1.09E-04	0.189883
	glucocorticoid metabolic process	CYP17A1, CYP11B2, HSD11B2, NR3C1	4	3.91E-04	0.680579
	steroid biosynthetic process	CYP17A1, CYP27B1, HSD17B2, HSD17B1, DHCR7, CYP11B2, HSD11B2, SRD5A1, HSD17B3, SRD5A2, NR0B1, CYP19A1	12	6.55E-08	1.14E-04
	Androgen and estrogen metabolism	STS, HSD17B2, HSD17B1, HSD11B2, SRD5A1, HSD17B3, SRD5A2, UGT2B7, CYP19A1	9	1.72E-05	0.020288
	inflammatory response	NOX4, ITGAL, RXRA, LYZ, NFKB1, TLR4, ADORA1, STAT3, PLAA, ALOX15, CNR2, HRH4, PLA2G7, LTA4H, ALOX5, PLA2G4B, F2R, AOC3	18	1.05E-05	0.018326
	regulation of inflammatory response	PRKCA, EDNRA, ADRB2, PTGS2, ELANE, PLA2G2A, TLR4, CALCRL, ADORA1	9	2.03E-05	0.035384
muscle tissue development	striated muscle tissue development	APP, MAPK14, RXRA, GAA, FKBP1A, CHRND, PPP3CA, TPM1, CDK5, CACNA1S, CHAT, F2R	12	2.06E-06	0.003585
	muscle tissue development	APP, MAPK14, RXRA, GAA, FKBP1A, CHRND, PPP3CA, TPM1, CDK5, CACNA1S, CHAT, F2R	12	3.34E-06	0.005822
	striated muscle cell differentiation	APP, NOS1, RXRA, PPP3CA, TPM1, CACNA1S, CHAT, F2R	8	3.95E-04	0.686758
	muscle organ development	APP, MAPK14, RXRA, GAA, FKBP1A, CHRND, PPP3CA, TPM1, CDK5, CACNA1S, CHAT, F2R	12	4.07E-04	0.708257
	muscle system process	KCNMA1, EDNRA, EDNRB, GLRA1, CAMK2D, GAA, FKBP1A, CHRND, TPM1, KCNQ1, CACNA1S	11	2.61E-04	0.455001
regulation of cell death	regulation of apoptosis	PTGS2, NFKB1, TLR4, NR3C1, ADORA1, SLC11A2, EDNRB, TUBB, APP, ERCC5, MAP3K5, GLO1, PRKCA, KCNMA1, SPHK2, RXRA, SPHK1, PIM1, ESR1, SMAD3, POLB, ESR2, CDK5, ADRB2, VCP, GSK3B, MPO, F2R, ALOX12	29	3.80E-05	0.066264
	regulation of programmed cell death	PTGS2, NFKB1, TLR4, NR3C1, ADORA1, SLC11A2, EDNRB, TUBB, APP, ERCC5, MAP3K5, GLO1, PRKCA, KCNMA1, SPHK2, RXRA, SPHK1, PIM1, ESR1, SMAD3, POLB, ESR2, CDK5, ADRB2, VCP, GSK3B, MPO, F2R, ALOX12	29	4.53E-05	0.078926
	negative regulation of apoptosis	SPHK2, SPHK1, ESR1, PIM1, SMAD3, POLB, NFKB1, ESR2, ADORA1, EDNRB, ERCC5, GSK3B, MPO, GLO1, F2R, ALOX12	16	3.64E-04	0.632638
	negative regulation of programmed cell death	SPHK2, SPHK1, ESR1, PIM1, SMAD3, POLB, NFKB1, ESR2, ADORA1, EDNRB, ERCC5, GSK3B, MPO, GLO1, F2R, ALOX12	16	4.20E-04	0.73015
	negative regulation of cell death	SPHK2, SPHK1, ESR1, PIM1, SMAD3, POLB, NFKB1, ESR2, ADORA1, EDNRB, ERCC5, GSK3B, MPO, GLO1, F2R, ALOX12	16	4.36E-04	0.757167
